# Considerations of private sector obstetricians on participation in the state led “*Chiranjeevi Yojana*” scheme to promote institutional delivery in Gujarat, India: a qualitative study

**DOI:** 10.1186/1471-2393-14-352

**Published:** 2014-11-05

**Authors:** Parthasarathi Ganguly, Kate Jehan, Ayesha de Costa, Dileep Mavalankar, Helen Smith

**Affiliations:** Indian Institute of Public Health Gandhinagar (IIPHG), Ahmedabad, Gujarat India; Department of International Public Health, Liverpool School of Tropical Medicine, Liverpool, UK; Division of Global Health, Karolinska Institute, NobelsVäg 9, Stockholm, Sweden; School of Nursing, Midwifery & Social Work, University of Manchester, Manchester, UK

**Keywords:** Public private partnership, Performance based financing, Private sector, Maternal health, *Chiranjeevi Yojana*, Demand side financing, Access to health care, Caesarean section (C-section), India

## Abstract

**Background:**

In India a lack of access to emergency obstetric care contributes to maternal deaths. In 2005 Gujarat state launched a public-private partnership (PPP) programme, *Chiranjeevi Yojana* (CY), under which the state pays accredited private obstetricians a fixed fee for providing free intrapartum care to poor and tribal women. A million women have delivered under CY so far. The participation of private obstetricians in the partnership is central to the programme’s effectiveness. We explored with private obstetricians the reasons and experiences that influenced their decisions to participate in the CY programme.

**Method:**

In this qualitative study we interviewed 24 purposefully selected private obstetricians in Gujarat. We explored their views on the scheme, the reasons and experiences leading up to decisions to participate, not participate or withdraw from the CY, as well as their opinions about the scheme’s impact. We analysed data using the Framework approach.

**Results:**

Participants expressed a tension between doing public good and making a profit. Bureaucratic procedures and perceptions of programme misuse seemed to influence providers to withdraw from the programme or not participate at all. Providers feared that participating in CY would lower the status of their practices and some were deterred by the likelihood of more clinically difficult cases among eligible CY beneficiaries. Some providers resented taking on what they saw as a state responsibility to provide safe maternity services to poor women. Younger obstetricians in the process of establishing private practices, and those in more remote, ‘less competitive’ areas, were more willing to participate in CY. Some doctors had reservations over the quality of care that doctors could provide given the financial constraints of the scheme.

**Conclusions:**

While some private obstetricians willingly participate in CY and are satisfied with its functioning, a larger number shared concerns about participation. Operational difficulties and a trust deficit between the public and private health sectors affect retention of private providers in the scheme. Further refinement of the scheme, in consultation with private partners, and trust building initiatives could strengthen the programme. These findings offer lessons to those developing public-private partnerships to widen access to health services for underprivileged groups.

## Background

Public-private partnerships are increasingly used in low and middle income countries (LMICs) to deliver a variety of health care and allied services. Although the ultimate responsibility of a country’s health system lies with its government, in countries with a diminished tax base, there are limits to what states can finance and achieve alone [1]. It is argued that effective stewardship implies a key role for governments as one of “oversight and trusteeship”, where the state “rows less and steers more” [1]. Collaborative partnerships, in which public authorities contract out services to the private sector, have been promoted as a realistic response to resource scarcity in the public sector in some contexts. In countries like India, where health care users face a choice between overstretched public systems or expensive and unregulated private services, successful public-private partnerships could harness the strengths and mitigate deficiencies of each sector. In India, public-private partnerships are seen as a pragmatic response to one of the most highly privatised healthcare systems in the world. Over two thirds of all health care expenditure is made in the private sector, mostly out-of-pocket [2, 3]. However, the evidence suggests that while some state partnerships with non-governmental organisations, voluntary organisations or the private for-profit sector have been successful, evidence remains mixed [4–6]. One reason for this is the private for-profit sector’s lack of enthusiasm for partnership with government, which they view with a degree of apathy and scepticism [7]. But owing to its widespread presence across the country, involvement of this sector is critical for implementation of Universal Health Care as declared by the Government of India [8].

Twenty percent of all global maternal deaths occur in India, and mostly among poor women [9]. Because most maternal deaths occur in the peripartum period, there is an assumption that a strategy based on birth in a facility equipped to provide skilled birth attendance and emergency obstetric care will lead to a reduction in maternal deaths [10]. In India, the main barrier to giving birth in-facility among poor women is financial access [11]. Removing financial barriers to facility birth and improving access to emergency obstetric care (EmOC) is imperative to reducing maternal deaths.

Health care provision in Gujarat is dominated by the for-profit private sector which provides most birthing facilities in the state, usually on a fee-for-service basis, paid out-of-pocket by the user. Weak obstetric care provision in the public sector [12] and wide availability of obstetric care in the private sector led the state Government to implement a public-private partnership to encourage poor women to give births in private obstetric care facilities. *Chiranjeevi Yojana (CY)*, or ‘Scheme for Long Life’ was launched in October 2005 to provide poor women access to emergency obstetric care in the private sector at no cost [12–14]. CY is a performance-based financing scheme, where maternity services are contracted out by the state to private obstetricians accredited on proof of certain criteria, such as providing a minimum of 15 beds and ready access to anaesthetic and blood transfusion facilities. The state pays accredited private obstetricians a pre-determined sum to perform facility births among poor women targeted by the scheme [12–14]. More than 800 private obstetricians joined the programme when it started (more than 50% of the total number of private obstetricians in the state) and nearly a million poor women have taken advantage of the scheme [15–17].

Recent evidence suggests Chiranjeevi Yojana is failing to attract and retain private doctors, with increasing attrition of obstetricians from the scheme. In the past five years, 50% of obstetricians are reported to have withdrawn their participation [17], yet little is known about why they have left, or what motivated them to join initially. Understanding this could lead to improvements in CY and better design and implementation of future public-private partnerships for widening access to services for underprivileged groups. In this study we explored the factors influencing private obstetricians’ decisions to enrol in the CY scheme, reasons behind their willingness or reluctance to continue, and the reasons why some choose never to participate at all.

## Methods

### Study setting

Gujarat is a state in the western part of India with a population of about 60 million. The percentage of the population living ‘below poverty line’ (BPL) is 16.7%, compared with 22% nationally [18], and the tribal population comprises 14.8%, with a significant overlap between these two groups. The Maternal Mortality Ratio (MMR) in Gujarat was 148 per 100,000 live births for 2007–2009, compared with the national MMR of 212 per 100,000 live births over the same period. Despite progress since 2004–2006, maternal health indicators in Gujarat still lag behind some states in the south of the country, for example Kerala where the MMR was 81 per 100,000 for 2007-2009 [19].

In Gujarat, as in other states in India, there is a chronic shortage of obstetricians in the public sector, and particularly in rural areas of the state [12]. Many factors influence the persistence of this gap, including low salaries, poor infrastructure, and few incentives for obstetricians to join government services in rural areas. Gujarat is more economically developed than other states, and three quarters of the 2000 registered obstetricians in the state work in the for-profit private sector [12]. Under these circumstances, where the state has a large and growing private health sector, a poorly functioning government sector, and a substantial proportion of the population eligible for social welfare programmes, an innovative scheme to co-opt the private sector to provide delivery care and access to emergency care seemed like a reasonable approach [12].

### Participants

The study was carried out in two purposively selected districts of Gujarat which were part of a larger parent study (MATIND), which recorded every facility conducting births in the two districts using GPS mapping. From this wider study, we had access to a comprehensive sampling frame of private practitioners. Each district is an independent administrative unit within the state, with an average population of two million. District 1 is more tribal (more than 20% of population) and less urban (11% of population), and district 2 is less tribal (1% of population) and more urban (27% population). The proportion of population without any work is marginally higher in district 2 [20].

From our comprehensive sampling frame, we selected facilities offering childbirth services within the two districts [21]. Study participants were qualified obstetricians whose facilities were eligible to participate in the CY scheme (able to perform Caesarean sections and transfuse blood). We selected obstetricians to capture variation in age, sex, location and their CY participation status (practitioners currently participating in the CY scheme, those who had discontinued their participation, and those who had never participated at all). Efforts were made to obtain a sample with a wide range of practitioner characteristics in order to identify what central, shared experiences around participation are common to each group, as well as areas of difference. Shortlisted private practicing obstetricians were identified and invited for interview by phone; they were informed of the purpose of the interview and estimated time required. Permission was sought to audio record the interviews and confidentiality was assured. Only three of those invited could not give us a suitable time for interview. We continued to interview until no new information was forthcoming, and reached saturation at 24 participants. This number represents about a third of the total eligible participants in these two districts (Table 1).

**Table 1:** **Participant characteristics**

Characteristics	District 1	District 2	Total
	N = 17	N = 7	N = 24*
Participation status at time of study	(%)		
Current participant	6 (35.3)	4 (57.1)	10 (41.7)
Discontinued	9 (52.9)	3 (42.9)	12 (50)
Never participated	2 (11.8)	0 (0)	2 (8.3)
Gender			
Male	13(76.5)	6(85.7)	19(79.2)
Female	4(23.5)	1(14.3)	5(20.8)
Location			
Large town, urban	7(41.2)	5(71.4)	12(50)
Small town, rural	10(58.8)	2(28.6)	12(50)
Age			
35 years or less	4(23.5)	2(28.5)	6(25)
More than 35 years	13(76.5)	5(71.5)	18(75)

*Note: Sampling frame.

District 1: Total private obstetricians: 55 (current participant: 16, discontinued: 34, never participated: 5).

District 2: Total private obstetricians: 21 (current participant: 8, discontinued: 12, never participated:1).

### Data collection process

We used semi-structured interviews to open up discussion about the obstetricians’ understanding of the scheme, their experiences of the CY scheme and what influenced them to participate, drop out, or never to participate at all, as well as their opinions about the scheme’s impact. The researcher arranged a mutually convenient time to interview those who agreed to participate; interviews were carried out at the obstetricians’ own facilities between July and September 2012. We piloted the interview with three doctors and made minor modifications to the topic guides. The first author of this paper (PG) conducted all interviews in a combination of the three languages commonly in use in Gujarat – Gujarati, Hindi, or English. On average the interviews took 40 minutes each, and were audio recorded; four interviewees did not wish to have their interviews recorded, so detailed notes were taken. The positionality of the interviewer (PG) was important in establishing a good rapport with the respondents; he is a physician with many years’ experience of working in close association with both private and government health sectors in Gujarat.

### Data analysis

A research assistant, present at each interview, transcribed all interview data and translated it into English for uniformity and the first author (PG) cross-checked a sample for accuracy. We used the Framework approach, a matrix-based method for ordering and synthesising data, to analyse the qualitative data [22]. Framework analysis is best suited to applied qualitative research, where the intention is to present themes identified in the data rather than develop or contribute to theory. PG coded all transcripts using a coding index based on concepts identified after reading and re-reading the transcripts. The team (PG, KJ, ADC and HS) discussed the initial grouping of the coded data into categories of related data, and from this identified the main themes and sub-themes through rounds of discussion. We frequently referred back to the raw data and used matrices containing data for each theme to help us identify similarities and differences across the different types of providers, age groups and gender.

### Ethical approval

This study has ethical approval from the Institutional Ethics Committee (IEC) of the Indian Institute of Public Health Gandhinagar. Written informed consent was obtained from those who agreed to be interviewed. Participant responses were anonymised during the analysis.

## Results

All of the obstetricians who participated in the study were owners of small private health facilities where they, as owner-doctors, made all decisions relating to clinical, technical, financial and administrative activities. Most practices we visited had 10–15 beds, and the main obstetrician employed both qualified and unqualified staff to work at the facility. More obstetricians were interviewed in district 1 due to the larger size of that district’s sampling frame and larger numbers of practising private obstetricians. The total number of obstetricians in district 1 was 55 compared with 21 in district 2, as mapped by the MATIND study team. Only two providers in our sample had ‘never participated’ in the scheme, and this could be due to the intense enrolment campaign across the state and political support the scheme enjoyed at the time it was launched. The gender distribution of our participants reflects the gender composition of obstetricians in Gujarat; there are fewer women obstetricians in the state (Table 1).

We identified six main themes that help to explain private practitioner decisions to participate in the CY scheme, the important influences on their decision making, and their experiences of participating in the scheme.

### Why should I participate? Tension between doing good for the public and making a profit

Private practitioners discussed at length the competing demands of providing a ‘public service’ to poor women, while at the same time needing to run a practice to make a profit. All the private practitioners we interviewed referred to making a significant initial investment in their practice and the expectation that this would translate into a reasonable profit:

*“Private [practice] is totally different. When someone has invested Rs. 7 million, he will try to make more money instead of getting involved in a loss making proposition. It is a business for him.” (Past participant, rural, male)*

At the same time many respondents, both currently participating and past participant groups, recognised and expressed the moral responsibility of a doctor to serve the poor. Private sector obstetricians claimed to work to their own kind of moral code, one which they believed would operate even in the absence of such schemes. They said it was common for them to treat poor patients and charge them less. As one doctor from an urban area who had discontinued participating in the scheme, claimed, ‘even when this scheme did not exist, almost every one of us was considering poor people’.

Some of the obstetricians, mostly in rural areas and currently participating in the scheme, commented that they were pleased that they could provide services free of charge to poor women while they themselves received reasonable fees through the scheme. A typical comment was that through the scheme doctors get paid by the government, but also get satisfaction of serving ‘poor people free of cost’.

Younger obstetricians observed a clear economic benefit to their participation in the scheme, particularly in the early stages of establishing their practice. One urban doctor who no longer participated in the scheme was certain that ‘new practitioners should definitely take [up] the scheme’. Others described how the scheme allowed them to launch their practices:

*“As a new comer I had enrolled into the scheme, and it helped me in my practice in the initial stage.” (Current participant, urban, male)*

*“We were struggling hard in our private practice and we got the chance, and it was the good chance to join the practice and to highlight ourselves […] Definitely benefited us to get name and fame.” (Current participant, rural, female)*

Private doctors from smaller towns felt that CY made more business sense in provincial or remote locations than in bigger cities:

*“The payment (Rs 2,800) what we get in CY is sufficient as the usual charge for normal delivery here is similar. In bigger cities, charges are around Rs10,000, so Rs 2,800 is very less for them.” (Current participant, rural, male)*

In spite of the scheme’s popularity among some younger practitioners, the majority of private obstetricians we talked to said the scheme was not economically viable, as the remuneration was inadequate, and it did not increase the volume of patients sufficiently to reap the benefits of ‘economy of scale’. One current participant in an urban area described his disappointment at the lack of new work the scheme brought, as initially he had thought that the ‘flow of patients would increase’. Other past participants also thought the scheme would ‘increase in the number of deliveries, patients will come to a private set up (facility), and we will get more work’.

In addition to disappointment over the volume of new patients the scheme would bring, a common concern was inadequate remuneration for complicated deliveries, such as those requiring blood transfusion or Caesarean section:

*“From this amount of Rs 2,800, medicines will cost us 1,000, anaesthesia cost 1,500. Hence doctor doesn’t get a single penny. That is why C.S. [Caesarean section] is not preferable to the doctor.” (Current participant, rural, female)*

Providers suggested that the cost of blood transfusion should be reimbursed separately, and that the Caesarean section rate calculated in the package should comprise at least 15–25% of total deliveries:

*“Amount of Rs 2,800 for normal [delivery] is alright but for C-Section it is quite less. Moreover C-Section rate is much higher [than 7% as calculated in CY] in private practice - around 20-30%.” (Current participant, urban, male)*

The disappointing volume of patients and the bundled remuneration for Caesarean sections and vaginal deliveries together meant that many obstetricians struggled with their (purported) desire to deliver a public good and their profit-making motives. As a consequence, a number of currently participating obstetricians’ spoke of this unresolved tension; those who had left the scheme cited it as a key reason why they were no longer participating in the scheme.

### Procedural burden discourages participation

Most doctors (current and former participants) complained of the scheme presenting a procedural burden to their daily practice. Past participants commented on the opportunity cost of engaging in the ‘considerable amount of paperwork involved to secure reimbursement’ and described long delays in getting the reimbursement. Both these factors appeared to be major reasons why some respondents discontinued their participation in CY:

*“Initially there was no problem, but later on the clerical work started increasing, so it was difficult to perform.” (Past participant, urban, male)*

*“When a pregnant lady comes to me for delivery, should I focus on saving the mother [and the baby] or on completing the form [documents]?” (Past participant, rural, male)*

A mistrust of the government health sector was evident in the way practitioners described their experiences of dealing with the local government authorities for reimbursement under CY. For example:

*“We have to give so many calls to them to get the payment. We have to meet them 2 to 3 times and I should go by myself to get the payment.” (Current participant, urban, male)*

*“When we go to the block office to get our payment, then the clerk behaves in such a way that [as if] he himself has to arrange payment from his pocket. They start picking up mistakes.” (Past participant, rural, male)*

For one doctor, a past participant working in an urban area, payment received for his services provided under CY came very late. On the day we interviewed him he remarked: ‘I got last year's payment [the] day before yesterday only’. When doctors sense such risks to their income, they may be tempted to compromise quality when treating CY patients as compensation. As an obstetrician who has never participated in the scheme remarked:

*“If the payment is less, quality gets compromised; disposables [may not be] used, low quality sutures used.” (Never participated, urban, male)*

One formerly participating obstetrician spoke of the positive outcome for his practice after discontinuing from CY:

*“Paperwork got reduced and income also [no longer] affected.” (Past participant, rural, male)*

Overall, perceived procedural burdens and delays were a considerable disincentive to continue or embark on participation in the scheme.

### Misuse of the scheme

The perceived misuse of CY by patients, health workers, government figures and other obstetricians alike was cited as a significant deterrent to private practitioners’ willingness to participate in the scheme. Almost all the obstetricians interviewed in both districts expressed their concern over misuse of the scheme by families they perceived as non-poor. According to the private obstetricians, many families who are not eligible manage to inappropriately procure a certificate of BPL (below poverty line) status from certifying authorities:

*“Many people who can afford the cost come as ‘BPL’ , but they have mobile, bikes, cars and everything […] All such persons are bringing [poverty] certificates from Talati [revenue office].” (Current participant, rural, male)*

*“People come in their Toyota car and show BPL cards.” (Past participant, rural, male)*

Moreover, many practitioners who had dropped out of the scheme told us that as misuse of CY by non-BPL women increased, they started to face a loss of revenue. This situation arose when the same clients who used to pay fees came in with appropriate BPL certification allowing them to access care for free:

*“Yes, as I have told you, the ones who were well off, started coming in the private hospitals pretending to be BPL […]”*

*[Interviewer] “The same people who used to come and used to make a payment?”*

*“Yes, and now they bring the BPL card and we started facing a loss.” (Past participant, rural, male)*

Another doctor concurred that the same was happening in urban facilities:

*“Same people who paid for first delivery now comes as BPL […] We lose earning. So you know when you feel that you are being cheated.” (Past participant, urban, male)*

Obstetricians were concerned about this ‘unfair procedure’ and considered it wasteful expenditure of government funds, diverting services from eligible families who actually need it.

Another source of discontent among the private obstetricians was what they considered the unfair means adopted by some of their fellow colleagues to profit personally from the scheme. A number of respondents described obstetricians paying community healthworkers to bring more patients to their hospitals. Respondents claimed that this included both ambulance drivers (of the state-supported 108 service) and Accredited Social Health Activists (ASHAs - voluntary community health workers paid an incentive to accompany poor women to facilities for hospital births). Conversely, providers gave examples of how some community health workers expected payment or favours from private practitioners for bringing women to the clinic. Providers we interviewed claimed:

*“Then Government promoted 108 [the ambulance service], and they [some private obstetricians] started pampering [bribing] 108 people.” (Past participant, rural, male)*

*“One ASHA offered me a CY form and said, ‘This lady has undergone home delivery, can you include her in your CY list [as institutional delivery for getting the payment]?” (Past participant, urban, female)*

*“ASHAs bring patients and expect some amount in return from us. I don’t entertain, so they send patients to other doctors who may do so.” (Current participant, urban, male)*

There was also evidence that some providers registered with CY exploit the scheme by accepting uncomplicated obstetric cases only. Practitioners located in rural as well as urban areas admitted that they tend to avoid accepting women who arrive late and with complications under CY, choosing instead to ‘push’ or ‘shift’ (i.e. refer) these cases to hospitals run by the government or charitable trusts. The reason commonly given for referring these women was that remuneration under CY is insufficient to cover the cost of blood transfusions or anaesthetist charges:

*“A CY patient comes to me with complication which needs C-Section. I am paid only Rs 2,800. You just tell me, can I manage it with this amount? No. So – there is a trend to…‘shift’. I will push that patient to somewhere else.” (Past participant, rural, male)*

*“Now as both types of delivery [normal and Caesarean section] have the same [remuneration] rate in the scheme, all the doctors try to go for normal delivery. Hence, neo-natal deaths are occurring more because of the long trial for normal delivery resulting in delay and foetal distress. This is a routine.” (Current participant, rural, female)*

Practitioners we interviewed also referred to misuse of the scheme by government workers. Private practitioners in urban and rural areas (both currently enrolled in CY and those who had dropped out), expressed displeasure at being placed under pressure from ‘influential (public) persons’ to accept their family members under the CY scheme. In most cases these requests were for individuals who were not eligible for CY, or who were not able to produce the necessary documents. Typically, practitioners explained they would receive a phone call to accept a particular woman and conduct the birth for free, and if they did not accept the woman, the influential caller ‘may create some problem’. Others believed they should not be ‘pressurised’ by the government authorities to accept their relatives under CY.

Related to their interactions with government officials, many practitioners in both districts, including current participants and those who had dropped out, described what they perceived sometimes as rude, uncooperative behaviour. Some of them also mentioned wilful delay at block level to make payments. One obstetrician said that some doctors are ‘giving [a] cut [a proportion of the payment to local officials] […] this should never happen’. Another went so far as to argue that without ‘some gratification’ to the system, working in the programme could be difficult. Officials could ‘pick up on mistakes’ for example, where forms were not filled out completely, and use this as a reason for delaying payment to the obstetrician.

### Going downmarket: participation in CY is perceived to lower private facilities’ status

In general, obstetricians who had a higher professional status (senior and established), social status (located in bigger cities than in remote areas) or financial status (charging higher fees) were less keen to participate in this scheme, which they felt was primarily a ‘poor people’s scheme’. As one senior obstetrician, a former participant from a rural area explained:

*“My charges are on higher side; Rs 4,000 in normal [delivery] and 8,000-10,000 for C-S [Caesarean section]. So CY is difficult for me.” (Past participant, rural, male)*

By drawing attention to his high fees, this obstetrician hints at what he sees as the incompatibility of his practice’s clientele with CY. Another former participant in an urban area seemed pleased that the status of his patients has changed since leaving CY:

*“Now, I am getting good socio-economically upper class people; when I was in CY, I used to get a lot of poor and village people.” (Past participant, urban, male)*

A younger, rural doctor explained that doctors in bigger cities were concerned about having to serve a poorer socioeconomic class of clients of CY as they thought that this might downgrade the image of their facility and deter ‘higher class’ patients who the providers said were the main source of income for them:

*“In bigger cities, certain hospitals have a stigma that they will not take this type of patient as they think that if such patients [CY beneficiaries] come to their hospital, the crowd of higher status patients will get [negatively] affected.” (Current participant, rural, male)*

The significance of status to doctors’ participation is also linked to peer decision-making. We observed that the decision of private obstetricians to either continue or discontinue their enrolment in the CY was greatly influenced by decisions taken by their peer group through the local branch of their professional body FOGSI (Federation of Obstetric and Gynaecological Society of India). In some towns, where the local branch of FOGSI had decided not to support the scheme, we found that almost all obstetricians had discontinued participation, whereas in other towns, almost all were continuing their participation:

*“Yes, I also think at times to leave this scheme but I am in this scheme just because of the competition, otherwise I would have left it because I have lost interest in it.”*

*[Interviewer]: “Competition means which type of competition?”*

*“Competition in the sense, see we have 10 to 12 gynaecologists in the same area, and three to four doctors have continued this scheme and other four have discontinued. So just to get more patients [not losing patients to them who continue enrolment in CY] we have continued to participate in this scheme.” (Current participant, urban, male)*

### Participating unwillingly: private sector perception of being coerced into taking on state responsibility

We found that private practitioners’ lack of trust in the government system adversely influenced the principle of partnership between government and private sector in CY. It was apparent in almost all the interviews across the three categories of respondents (those who were currently participants, past participants and had never participated). The providers were united in their opinion that rather than making government health staff and facilities accountable for maternal health services, the CY scheme ‘tends to pass on this responsibility to the private sector’. In addition, it was their view that private practitioners ‘shoulder the risk of providing these services’ with no additional legal protection provided by the government in case they are faced with maternal deaths or severe complications. This seemed to influence participation in the scheme, with some doctors dropping out and others avoiding complicated cases, because they felt they would expose themselves to potential litigation without any government support:

*“Complicated cases are avoided by us because if there is any mortality then it becomes very difficult for any doctor. Government has told us that it is our responsibility to tackle those matters; whether it is criminal, civil or consumer. Only the burden is on doctors, no protection at all.” (Current participant, rural, female)*

*“I don’t take complicated CY cases because of fear that I will be held responsible if any unwanted things happen. Whole responsibility comes on us only. Government should give full protection in case of complications.” (Past participant, rural, male)*

Older practitioners and those who had dropped out of the scheme were critical of what they perceived as unwarranted government enquiries into private practices under the CY scheme, and the negative publicity this could involve. They commented that the government seemed ‘suspicious’ of private practices, and were quick to report any malpractice or misconduct, yet ignored the irregularities in government hospitals:

*“Government does not bother for mistakes done by Government staff but in case of private doctors they are very strict. They are always suspicious about private doctors.” (Past participant, urban, female)*

*“Some colleagues faced enquiries - demoralising and bad for social and professional reputation.” (Never participated, urban, male)*

Many practitioners perceived there to be an association between participation in CY and their vulnerability under the Pre-Natal Sex Determination Techniques (PNDT) Act. The act is in place to prevent female foeticide which is a major social problem leading to a disproportionate sex ratio in the population. Under the act, obstetricians are forbidden to tell parents the sex of the child during an ultrasound examination. Obstetricians feared that if they did not participate in CY, government officials would accuse them of violating the PNDT Act, or not grant them registration to carry out ultrasound examinations. Not surprisingly, practitioners who had dropped out of the scheme were more willing to talk about this, whereas those remaining in the scheme raised the same issues but were more guarded in their descriptions:

*“They told, ‘You have to join otherwise we will not give you registration for sonography’…yes it was [a] threat, by the Collector.” (Past participant, rural, male)*

*“Government people keep us threatened that action may be taken against us in the pretext of some irregularity in PNDT.” (Past Participant, urban, male)*

### Participation in CY perceived as a risk by the private sector

Another theme we identified in the data, which is closely linked to other risks raised by the participants, is their perception of the clinical risks associated with participating in the scheme. There was a clear sense that clinically difficult cases cluster in CY because of the socioeconomic background of the beneficiaries, and this deterred providers from participating. Private practitioners in rural areas in particular felt that CY beneficiaries as a group are at much higher risk of complications, as they are often highly anaemic, malnourished and multiparous. For example, this urban practitioner, a past participant, explained:

*“There are differences between BPL and APL [Above Poverty Line] patients. Complications are high among BPL patients - anaemia, sepsis, unhygienic conditions. Anaemia is a big problem indeed.” (Past participant, urban, female)*

Obstetricians commented that CY beneficiaries often arrive late to the facility, already in established labour. At the same time, few BPL patients attend free antenatal care. Without knowing the detailed history of the pregnant women, practitioners were of the view that they are unable to anticipate complications, or prevent them by treating underlying causes such as anaemia or malnutrition. As two past participants from rural areas stated:

*“Three to four ANC [antenatal care visits] is a must, but most patients don't come for ANC; [they] directly come for delivery.” (Past participant, rural, male)*

*“Here people receive two TT [Tetanus Toxoid] from the sister (community worker) and think their ANC is finished.” (Past participant, rural, female)*

The pressures placed by a large volume of high risk patients led some obstetricians to question the ability of doctors to offer sufficient quality care. Several practitioners raised concerns about the quality of care for women during delivery at private facilities participating in the scheme, with one provider confident that, under such circumstances ‘there are some people who compromise quality’. Some of the older practitioners we interviewed (those who had dropped out or had never participated), suggested that many clinical procedures including delivery were conducted not by the doctor, but by ‘unqualified staff’ in many private hospitals. This was perceived not as a deliberate misuse of the scheme, but as a response to resource shortages:

*“How a private practitioner can manage himself to see 100 patients, do 5–7 deliveries with 1–2 C-Sections in a day? In reality, s/he entrusts 5–6 nonqualified person (trained by him/her) to do a lot of things, including deliveries. So, where is the quality?” (Past participant, rural, male)*

Many practitioners, old and young and across all categories (participating, dropped out and never participated in CY) stated that the Caesarean section rate is relatively high in private practice because it is the preference of the practitioner to do elective surgery in cases with complications. Private obstetricians said that they usually avoid a trial of normal labour and delivery as it increases the chance of emergency Caesarean delivery, which is always challenging in single doctor practices without ready availability of blood and an anaesthetist:

*“Now here we are, single handed practitioner, and without teamwork; to tackle such emergency is difficult.” (Past participant, urban, male)*

*“In private practice it is difficult to tackle emergency C-section as blood, and an anaesthetist may not be readily available.” (Past participant, urban, male)*

## Discussion

In this study we have documented, through carefully conducted interviews, some of the factors that have influenced private obstetricians’ decisions to participate, not participate or withdraw from a state led private sector initiative to increase institutional deliveries among poor women in Gujarat state. Providers still participating, and those who recently dropped out spoke about a tension between doing public good and making a profit. They expressed a need to balance altruism with entrepreneurship, and were disappointed when the scheme did not yield an increase in new patients, or provide sufficient remuneration for complicated deliveries. On the other hand providers told us that informally they feel they do behave charitably towards poor patients regardless of the scheme, by treating poor women in their practices and charging them less. Besides the procedural burden associated with participation in the scheme, our findings show that eligible practitioners are discouraged from participating because they perceive that accepting poor women as clients will damage the reputation of their facility, and that dealing with the clinical complications associated with this group of women was too much of a risk. We also identified a lack of trust between the private and public sector, with private practitioners sceptical of the rationale for participating in the scheme, and deterred by the potential and real misuse of the scheme by non-eligible families.

### Why do private practitioners enrol and continue their participation in the CY scheme?

Some of the obstetricians’ claims of a personal ‘moral code’ driving them to participate and to provide public services to the poor has been noted in another small study in Gujarat state (Ranjan P: A study on factors for and against participation in the Chiranjeevi scheme by private sector obstetricians, unpublished). This self-declared motivation of altruism is consistent with the ethical code of the Indian Medical Act, to which all Indian doctors are required to adhere [23]. At the same time however, they described a parallel incentive in joining the scheme: wanting to build a business, or establish their reputation. This derives from a different kind of motivation, where health care services are viewed as a ‘market good’ [24]. Other reports on the CY scheme also describe a similar observation [13]. From this viewpoint, the provision of health care is an entrepreneurial activity, arguably a view that runs counter to current policy emphasis in India of Universal Health Coverage [25]. With India’s health system in financial crisis, current debate asks how realistic this notion of ‘health as a public good’ is, or even how ‘Indian’ [26]. Rather than second guess practitioners’ ‘true’ motivations, our concern here is whether private obstetricians’ and the state’s interests overlap with regard to the CY. With any public-private partnership, it is not necessary that partners share exactly the same interests, only that at some point they converge to make participation for both an attractive prospect. While the state’s interest was to increase access to birth in a private facility for poor women, a part of the private obstetric sector saw the programme as a means to build their own practices, increase patient volumes in their facility and so were motivated to join the programme as they had a shared interest with the state in implementing the CY. Not all providers shared this motivation, but it is likely that those who did were more likely to join and remain in the programme.

### Why have private practitioners left the scheme, or never joined?

The doctors interviewed voiced a number of complaints which they claimed left them dissatisfied and prompted their withdrawal, or deterred them from ever participating. Chief amongst these was the notion that the scheme is not economically viable. Insufficient remuneration for complicated deliveries was cited as a key cause, with a flat-rate pricing structure (covering an estimated percentage of Caesarean sections) unpopular with many doctors. Based on evidence that differential pricing structures cause inflation in Caesarean sections [27, 28], the scheme designers attempted to embed this disincentive for unnecessary surgical intervention. According to doctors interviewed in this study, this is likely to be at root of much provider attrition. Other authors also argue that though the remuneration package was decided in consultation with different stakeholders [12], private providers did not clearly understand the concept of remuneration based on a fixed fee for 100 deliveries, instead thinking in terms of each individual case [29]. The payment for surgical deliveries has recently been doubled in Chiranjeevi [30], though the impact of this on attracting doctors back to the scheme is not yet clear. As our study findings suggest that the scheme is most popular with two main groups of doctors – those starting out in practice and needing to establish a reputation, and those in rural areas with little competition – it is probably more efficient for the scheme to target specific groups of doctors within the private sector, where there is a clear convergence of state and private sector interests.

Another reason doctors withdrew from CY was the amount of paperwork and procedural burden generated by the scheme. Key issues for attention here concern how to balance the need to monitor such schemes, whilst streamlining the paperwork and reducing perceived bureaucracy. The systems deployed by other schemes such as the Indian government’s *Rashtriya Swasthya Bima Yojana* (RSBY), which some respondents seemed to prefer to CY, need to be explored [31].

A unique observation in our study is a pattern of discontinuation; in some towns a large number of private obstetricians had dropped out of the CY scheme, and in others a large majority remained enrolled. A possible explanation for this is that the decision to continue or discontinue was taken as a group, rather than by individual practitioners. Practitioners told us that participation is discussed in the local branch of the professional body (FOGSI) and a uniform decision is often taken to discontinue from CY. This was apparently driven by business sense and financial profits. Unless all the obstetricians of the town discontinued en masse, there was a chance that individual practices could lose out to those practitioners still enrolled. This process of joint decision-making should be taken into account at the planning, promotion and implementation phases of such schemes.

The providers’ misgivings about bureaucracy are closely linked to the lack of trust the private providers perceive in their dealings with the government. Providers voiced concerns about the power balance within the partnership, and the notion that the government monitors the private sector much more closely than it monitors itself. Concern about contract management and scheme monitoring by government officials was mentioned in another study [29]. This sense of asymmetrical power sharing breeds feelings of resentment from the private doctors and fuels mutual suspicion and mistrust. Feelings that the private sector is thus the ‘little partner’ are compounded by the doctors’ sense that the public sector might be shirking responsibility (and landing the private sector with all the risk). Trust is further eroded by the perceived threat that some government officials use the PNDT act to coerce providers to participate in the scheme. In addressing the trust in a public-private partnership, the difficulty lies in achieving the balance between the need for some government control, and the need for trust. Other literature has mentioned that the sharing of vision, values and an atmosphere of trust are key factors for any successful partnership [7].

Reported widespread misuses of the scheme–by target beneficiaries, workers in the health system, government officials and by obstetricians themselves–further discouraged obstetricians from participating in CY. Many practitioners claimed to find a drop in their revenue when a large number of (previously fee-paying) clients began claiming free services under the scheme, and others gave examples of non-poor persons holding legal poverty certificates, which they used to access scheme benefits. Though intangible, trust is important and its presence or absence has tangible results. Building societal trust is likely to begin to tackle corrupt use of such schemes.

Senior obstetricians in bigger cities, with higher fees and serving ‘higher class’ clientele, who considered themselves ‘high profile’ providers, were concerned with the lack of business sense in the scheme, and felt it somewhat below their ‘status’ to treat poorer patients. Their apprehension, that higher class clients (a source of major income to them) may dislike visiting their facility alongside CY patients, discouraged their participation in the scheme. By contrast, the smaller town obstetricians, with lower fees and no such sense of stigma about CY clients, did not share this view. This observation in our study is in contrast to earlier suggested findings that CY was perceived by senior providers as a charitable venture, rather than a PPP [32]. It again points to the need for more targeted promotion of the scheme among suitable providers.

This need for targeted promotion extends to another serious concern, that obstetricians sometimes find ways to maximise profits out of the reimbursement package, some actively avoiding complicated cases which require more costly delivery, blood transfusion or longer hospital stays. Other research has also highlighted this profit maximising behaviour among private providers, and ‘cherry picking’ of uncomplicated cases [29, 32]. The result is that high risk cases are passed back to the public sector or in any case, moved out of the programme. Other cost cutting measures reported included using inferior equipment, supplies and medication as well as inducing ASHAs or 108 ambulance staff to bring more eligible women to their practice. Together, these profit maximising activities all have an impact on the quality of care that women receive at private facilities enrolled in CY. Ultimately, encouraging women to give birth in facilities under these circumstances is unlikely to reduce maternal and neonatal morbidity and mortality.

This raises questions over the danger of poor women ‘falling through a gap’ , either receiving poor quality care from CY providers dissatisfied with their remuneration, being turned away from providers disinterested in joining the scheme, or being left with no choice beyond the poor quality provision in a struggling public sector. This would negate the purpose of CY, which is to enable poor women to access functioning maternity services. The threat of this vision underlines the importance of making this scheme and others like it attractive to private providers, which policymakers and scheme designers can do, by examining the causes of attrition from PPPs such as these. The recommendations we draw from this study from which similar schemes and interventions could benefit are several, and are reiterated here.

One, such schemes must be streamlined and devoid of lengthy bureaucratic procedures that waste providers’ time (this includes timely repayment schedules). Two, the reimbursement package must be attractive enough to providers to enable them to run their businesses and provide quality services, without fears of failing to do the former compromising the latter. Three, time and resources spent attempting to coerce reluctant providers into joining the scheme is wasted, resulting in half-hearted delivery of services. As our study found in this context, junior doctors starting out in their practice and doctors in rural areas (who face less competition for patients) find the scheme more suited to their aims and objectives, and are more likely to deliver the scheme satisfactorily. Four, the potential for misuse of the scheme by beneficiaries and others (which repels providers’ participation) must be minimised. The thorny issue of misuse can be approached through improved targeting, though there is, as yet, no panacea to preventing corruption from all stakeholders involved in schemes such as these. Five, more transparency between sectors is required to build trust, which is critical for any partnership to work; schemes similar to CY need to establish good relationships between public and private sectors. This would enable strong governance and allow the state to monitor and regulate partners, without resistance or suspicion on either side. Poor leadership, weak contracts and poor monitoring are significant threats to the success of programmes such as this. The need for trust relates to six – the perceived need among providers for some form of risk-sharing in serving higher risk patients. The perception that difficult cases cluster around CY and that little protection is offered for private providers in the scheme was an important concern, and is a disincentive for providers to join. As this is a partnership, it is not unreasonable to expect some of the risk to be shared, which would improve attrition rates according to providers interviewed in this study.

Lastly, the existence of PPPs does not preclude efforts to strengthen the public sector; a strong public sector remains a key goal in India’s vision for universal health coverage. PPPs, while imperfect, can be regarded as an interim measure to provide a safe birth environment for poor women. Where public sector provision has failed to deliver safe services and shows little sign of doing so soon, who delivers the services should be less important than how, and how well, they are delivered.

### Strengths and limitations

We conducted interviews in just two districts of Gujarat state, but these were carefully chosen to allow us to explore the views of private practitioners in a rural and an urban context. Our study was conducted as part of a larger programme of work funded by the EU, which included comprehensive GPS mapping of private practitioners in the state. This enabled us to confidently select from this list of qualified obstetricians, those whose facilities were eligible to participate in the CY scheme. We selected obstetricians carefully to include men and women of different ages and participation status (practitioners currently participating in the CY scheme, those who had discontinued their participation, and those who had never participated at all). Based on the principle of maximum variation, this sample allowed us to identify the central, shared experiences around participation common to each group, as well as areas of difference. We also selected respondents from different geographical areas: those which were larger and urban, smaller and urban, predominantly rural, peripheral, remote and/or tribal to gain a wide range of contexts. Another strength is that the principle investigator who conducted the interviews in this study is a practising public health physician with experience working in the public and private sector in Gujarat state; this positionality allowed him to quickly establish rapport, generate discussion, and gather in-depth and frank accounts of the CY scheme.

When designing the study, we anticipated that providers would refuse to be interviewed or allow interviews to be audio recorded, due to concerns about confidentiality. Fortunately, only three obstetricians we contacted did not agree to the interview, primarily due to time constraints, and only two respondents interviewed expressed some reservation about recording. These participants made a few comments ‘off the record’ at the end of the interview, which were noted down by the interview team. We feel that overall quality of the data has not been compromised. An important point to observe in studies such as these however is the potential for social desirability bias, particularly where providers discuss their self-declared altruism. We cannot discount the possibility of such bias entirely and we have taken this into account into our analysis.

## Conclusion

Exploring private practitioners’ views of the CY scheme, the scheme’s functioning, and its ability to increase access to CEmOC among the poor and tribal populations is timely. Our findings suggest that private practitioners share some common concerns about participating in this performance-based financing scheme. The retention of private providers is compromised by procedural and remuneration problems, leading providers to perceive it as an unprofitable proposition. Procedural burden coupled with misuse of the scheme at different levels of the health system prompted private providers to feel dissatisfied with their participation in the scheme. Some doctors had concerns about the quality and accountability that could be provided under the financial constraints of the scheme. The data from this study suggest that an emphasis on strong contractual agreements which are well-monitored by the state and allow better negotiation with private providers would increase success of the scheme. The scheme is under review by the state government and these findings could help improve its design and likelihood of impact on maternal and neonatal mortality and morbidity.

### RATS guidelines

We declare that our research has adhered to the guidelines for Qualitative research review guidelines (RATS) prescribed by BioMed Central.

## Abbreviations

ASHA: Accredited Social Health Activists
BPL: Below poverty line
CEmOC: Comprehensive Emergency Obstetric Care
C-section: Caesarean section
CY: *Chiranjeevi Yojana*
EU: European Union
FOGSI: Federation of Obstetric and Gynaecological Societies of India
GPS: Global positioning system
LMIC: Low and middle income countries
MMR: Maternal Mortality Ratio
PNDT: Pre-conception and Pre-Natal diagnostic techniques
PPP: Public private partnership
RSBY: *Rashtriya Swasthya Bima Yojana* .
